# Mortality Trends Due to Skin Melanoma in Poland in the Years 2000–2020

**DOI:** 10.3390/ijerph192316118

**Published:** 2022-12-02

**Authors:** Elżbieta Dziankowska-Zaborszczyk, Irena Maniecka-Bryła, Małgorzata Pikala

**Affiliations:** Department of Epidemiology and Biostatistics, Social and Preventive Medicine, Medical University of Lodz, Żeligowskiego 7/9, 90-752 Łódź, Poland

**Keywords:** skin melanoma, mortality, CDR, SDR, joinpoint trends, APC, AAPC, Poland

## Abstract

The aim of this article is to assess mortality trends due to skin melanoma in Poland between the years 2000 and 2020, taking into account gender and place of residence (urban, rural). The subject of the analyses was data on 25,061 deaths that occurred between 2000 and 2020 due to skin melanoma (C43 according to ICD-10). Mortality rates due to this cancer, both crude (CDR) and standardised (SDR), were calculated. Trends on the calculated rates were analysed using the annual percentage change (APC) and average annual percentage change (AAPC), obtained from joinpoint regression models. Over the study period, the standardised death rate (SDR) due to skin melanoma in Poland increased from 3.60 to 4.03 per 100,000 population (AAPC = 1.1; *p* < 0.05), for urban residents it increased from 3.56 to 3.91 (APC = 1.2; *p* < 0.05) and for rural residents it increased from 3.00 to 4.24 (APC = 2.2; *p* < 0.05). A higher growth rate in terms of the SDR value between the years 2000 and 2020 was recorded in men compared to women and in rural when compared to urban residents. In Poland, mortality due to skin melanoma is on the rise. The early diagnosis of this cancer should become common practice in the Polish population.

## 1. Introduction

The most common skin cancers are basal cell carcinoma (BCC), squamous cell carcinoma (SCC) and melanoma (malignum melanoma—MM).

Melanoma, being a neoplasm, originates from pigment cells of the skin (melanocytes). It occurs on the skin and in the mucous membranes of the mouth and genital organs as well as in the eyeball. Skin melanoma accounts for more than 90% of all melanoma cases [1]. Among the most common cancers worldwide, skin melanoma occupies the 19th position [2].

Skin melanoma is one of the most malignant skin cancers and has the worst prognosis. It is characterised by early metastases and low sensitivity to cytostatic treatment, which results in high mortality. It most cases, it metastasises to surrounding lymph nodes, the lungs and brain. Skin melanoma most commonly develops from existing skin lesions, pigmented nevi, but it can also form on healthy skin.

The most common locations for this tumour are, in men, the trunk and head (41% and 23% of cases) and, in women, the lower and upper limbs (38% and 25% of cases) [3].

The main risk factors for skin melanoma include: exposure to UV radiation—both sun and tanning beds, sunburn, skin phenotype (skin colour, hair colour, eye colour), the presence of nevi (especially congenital nevi), immunosuppressive treatment, genetic background and a positive family history [4,5,6,7,8]. According to estimates, UV radiation (both solar and from tanning beds and lamps) is responsible for 80% of skin cancers worldwide [9], and solar radiation alone is responsible for at least 65% of the occurrence of skin melanoma [10]. There is growing evidence that the occurrence of MM is associated with the use of sunbeds and tanning lamps, especially before the age of 30 [11]. The geographical latitude of residence is also considered a risk factor for skin melanoma [7]. Age and gender are the best-known non-modifiable risk factors for this type of cancer. The risk of developing the disease increases with age, and the incidence of melanoma in people under 40 years of age is higher for women than men. For those above 75 years of age, the incidence is almost three times higher in men than in women [4]. 

Almost 85% of MM cases occur in developed countries. Skin melanoma ranks the 6th most commonly diagnosed cancer there [12]. 

The incidence of skin melanoma is increasing faster than the incidence of any other malignancy [1]. In just one decade, the number of MM cases has increased by more than 40%. In 2008, nearly 200,000 cases of skin melanoma were confirmed worldwide, and in 2018, there were already more than 287,000 new cases [2,13]. 

The highest melanoma incidence rates are observed in Australia (37/100,000) and the lowest ones in South-East Asia (0.2/100,000) [1]. 

An increase in the incidence of skin melanoma results in an increase in the number of deaths. Globally, 46,000 people died from MM in 2008 and nearly 61,000 in 2018 [2,13]. 

In Poland, in 2019, skin melanoma was the 10th most common cancer observed in men, the 11th most common cancer observed in women and the 14th and the 16th most common malignant neoplasm contributing to mortality in men and women, respectively [14]. 

According to data from GLOBOCAN estimates, in 2020, new cases of skin melanoma in Europe accounted for more than 44% of all cases of this type of cancer worldwide. At that time, deaths due to skin melanoma in Europe accounted for 47% of all deaths worldwide. In 2020, nearly 145,000 new cases of MM were reported in Europe, including more than 26,000 in Central and Eastern Europe. In the same year, 27,000 deaths due to skin melanoma were reported in Europe. Of this number, 9000 cases were noted in Central and Eastern Europe [15]. In the year 2020, in Poland, 1427 skin melanoma-related deaths were reported [16]. These accounted for more than 5% of deaths in Europe and nearly 16% of deaths in Central and Eastern Europe. 

In Europe, skin melanoma accounts for less than 5% of all skin cancers and is responsible for more than 80% of skin cancer deaths. In this region of the world, deaths due to MM account for 1–2% of all malignant cancer deaths. The incidence of this cancer in Europe is higher among women than men. The highest incidence rates of skin melanoma are observed in northern and north-western European countries, namely Norway, Sweden, Ireland, the UK, the Netherlands, Germany and Switzerland, and the lowest in Spain, Portugal and Greece [17,18]. The highest mortality rates are noted in Scandinavian countries and the lowest in Greece [19,20]. 

Trends in melanoma incidence and mortality vary across European countries. In Northern Europe, where incidence rates are highest, decreasing trends in both incidence and mortality have been observed [21].

People with fair skin prevail in Poland; approximately 3000 people are diagnosed with skin cancer annually. Skin melanoma accounts for just over 2% of all diagnosed cases of malignant neoplasms and causes 1.4% of cancer-related deaths. With regards to mortality from malignant tumours, skin melanoma ranks the 15th among men and 16th among women [22]. The incidence of skin melanoma in Poland is almost twice as low as the average incidence for 27 EU countries [23], and mortality due to this cancer is approximately 20% higher than the EU average value [24]. In Poland, the number of deaths due to skin melanoma has been increasing in recent years; in 2000, there were 928 deaths from this cause, and in the year 2020, the number was 1427.

## 2. Materials and Methods

The study material included data from 8,469,294 death certificates, obtained in Poland between the years 2000 and 2020. The data were made available by the Central Statistical Office. Poland is a country with 100% completeness in terms of death registration.

During the analysed period, 1,969,155 people died in Poland due to malignant tumours, including 25,061 due to skin melanoma. Deaths due to this neoplasm accounted for 0.3% of all deaths and 1.3% of deaths due to malignant neoplasms. Death due to skin melanoma was identified on the basis of code C43 according to the International Statistical Classification of Diseases and Health Problems, Revision 10 (ICD-10), entered in the death certificate as the primary cause of death.

The collected material allowed for the calculation of crude (CDR) and standardised (SDR) death rates per 100,000 people for each year. These rates were calculated for the total population of Poland as well as for the urban and rural population, considering the gender for each place of residence of the deceased.

Death rates for particular years were calculated according to the following formula:

$$CDR=\frac{k}{p}\cdot 100,000$$

where *k* stands for the number of deaths due to skin melanoma and *p* is the size of the population in a particular year. The data regarding the population size and information regarding urban and rural areas were obtained from the Central Statistical Office; urban areas were classified as covering areas located within the administrative borders of cities and rural areas covered areas beyond the administrative borders of cities [25].

Among the 25,061 deaths due to malignant melanoma recorded in Poland in 2000–2020, 15,771 (62.9%) occurred among urban residents and 9290 (37.1%) among rural residents.

Death rates were standardised using a direct method based on the European standard population, updated in 2012 [26]:

$$SDR=\frac{\sum_{i=1}^{N}\frac{k_{i}}{p_{i}}\cdot w_{i}}{\sum_{i=1}^{N}w_{i}}$$

where *k_i_* is the number of deaths due to skin melanoma, *p_i_* is the population size in the *i*-age group, *w_i_* is the weight of the *i*-age group, derived from the standard population distribution, and *N* is the number of age groups.

Time trends were assessed based on results of joinpoint regression models, obtained with the use of the Joinpoint Regression Programme, a statistical package developed by the U.S. National Cancer Institute for the Surveillance, Epidemiology and End Results (SEER) Program [27]. The joinpoint regression method is an extended variation of the linear regression y = a + bx, where: y = ln(z), x is the year and z is the value of the death rates calculated in the study. In the joinpoint regression method, the time trend is expressed by a broken line consisting of straight lines (segments) joining at joinpoints. At these points, a statistically significant (*p* < 0.05) change in trend is observed. The test used to assess the significance of trend changes utilises the Monte Carlo permutation method [28]. The annual percentage change (APC) was calculated for each broken line segment, and the average annual percentage change (AAPC) was calculated for all years of the analysed period, with corresponding 95% confidence intervals (CI). 

The calculated values for APC and AAPC for death rates due to skin melanoma between 2000 and 2020 enabled the changes in mortality trends due to this cancer over the analysed period of time to be assessed. If there were no statistically significant changes in the rate and/or direction in the values of the calculated rates during the study period (number of joinpoints = 0), the equation AAPC = APC was adopted.

## 3. Results

**Figure 1:**
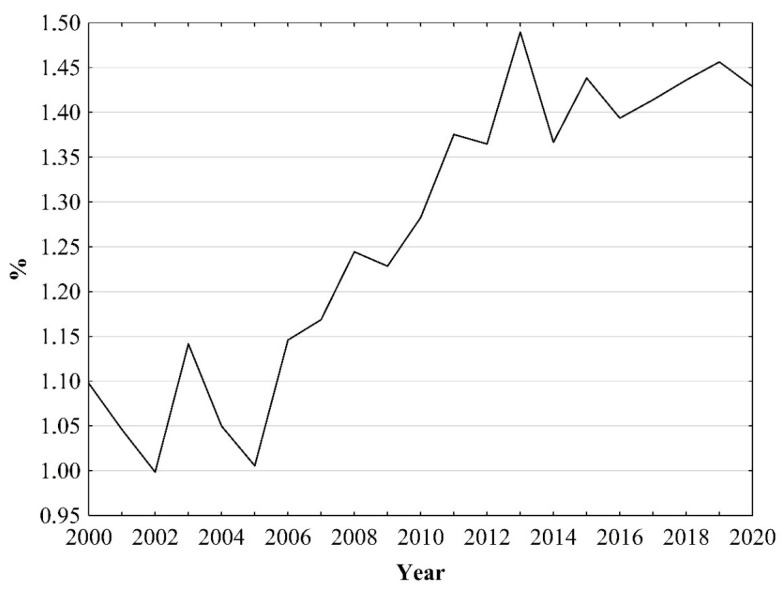
Percentage of deaths due to skin melanoma in comparison to deaths due to malignant neoplasms in Poland in 2000–2020. Source: own calculations.

In the years 2000–2020, 25,061 people died due to skin melanoma in Poland. During this period, the proportion of deaths due to this cancer in relation to the total number of deaths due to malignant tumours increased from 1.1% in 2000 to 1.43% in 2020 (Figure 1).

**Figure 2:**
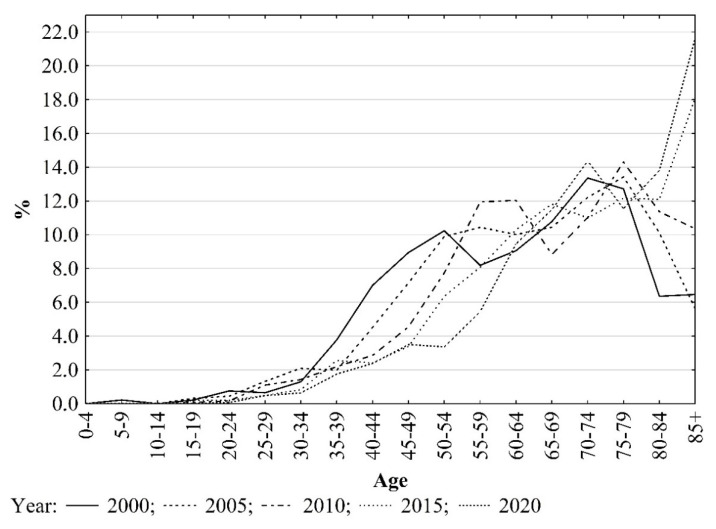
Deaths due to skin melanoma in Poland in selected years by age. Source: own calculations.

In the first two decades of the 21st century, the average age of people who died of skin melanoma in Poland increased from 62.6 in the year 2000 to 72.0 in the year 2020 (in the male group, this increased from 61.5 in 2000 to 69.6 in 2020, and in the female group, it increased from 63.7 in 2000 to 75.0 in 2020). The mean age of men who died of skin melanoma was lower than the mean age of women. Corresponding changes were also noted for urban and rural residents, and the mean age of male rural residents who died of this cancer was lower than that of male urban residents. The mean age of female rural residents was higher than that of female urban residents (Table 1 and Table S1). The increase in the mean age of people who died due to skin melanoma was associated with an increased proportion of deaths of older people, i.e., those aged 75 years and over, in the total number of deaths due to this cancer (Figure 2).

The number of deaths due to skin melanoma in Poland during the analysed period increased from 928 in 2000 to 1427 in 2020. The increase in the number of deaths due to this neoplasm was observed for all inhabitants of Poland as well as for urban and rural residents and for men and women. A higher number of deaths was recorded for men (13,062; 52.1%). A vast majority of deaths due to skin melanoma occurred in urban residents (15,771; 62.9%). At the beginning of the study period, between 2000 and 2004, the number of deaths due to MM was higher in women than in men. Since 2004, the situation has been different—the number of deaths due to this neoplasm has been higher in the male subpopulation than in the female subpopulation across Poland as well as in urban and rural residents (Table 2, Table 3, Table 4 and Tables S2–S4).

Skin melanoma-related mortality increased with age (Table 5). From 2000 to 2020, in Poland, the values of death rates per 100,000 population were on the increase. In 2000 and 2020, the crude death rates (CDRs) calculated per 100,000 population were 2.43 and 3.73, respectively. During this period, the annual growth rate of CDR values was 2.8% (APC = 2.8; 95% CI: 2.4–3.2; *p* < 0.05). Both male and female groups demonstrated a noticeable increase in death rates: from 2.50 to 4.11 (APC = 3.2; 95% CI: 2.7–3.7; *p* < 0.05) in the male group and from 2.36 to 3.38 in the female group (APC = 2.4; 95% CI: 2.0–2.9; *p* < 0.05) (Table 2, Table 3, Table 4 and Table 6).

With regards to urban residents, the CDR values were 2.57 in 2000 and 3.88 in 2020 (APC = 2.8; 95% CI: 2.3–3.3; *p* < 0.05); for men, these values were 2.76 in 2000 and 4.34 in 2020 (AAPC = 3.1; 95% CI: 2.0–4.3; *p* < 0.05), whereas for women, they were 2.40 in 2000 and 3.47 in 2020 (APC = 2.4; 95% CI: 1.8–3.0; *p* < 0.05). As for male urban residents, the annual growth rate of CDR values changed significantly in 2011. From 2000 to 2011, death rates related to skin melanoma were relatively high, with an annual increase rate of 4.4 % (APC = 4.4; 95% CI: 2.9–5.9; *p* < 0.05), whereas between 2011 and 2020, the growth rate of CDR values decreased to 1.6% (APC = 1.6; 95% CI: −0.4–3.6; *p* > 0.05) (Table 2, Table 3, Table 4 and Table 6).

For rural residents, the CDR values contributed by skin melanoma were 2.19 and 3.50 (APC = 2.9; 95% CI: 2.4–3.3; *p* < 0.05) in the years 2000 and 2020, respectively. For men inhabiting rural areas, the CDR values in 2000 and 2020 were 2.09 and 3.78 (APC = 3.2; 95% CI: 2.7–3.7; *p* < 0.05), respectively. With regards to female rural residents, the death rate value related to skin melanoma increased from 2.29 in 2000 to 3.23 in 2020. The annual growth rate of CDR values in the female group throughout the entire analysed period was 2.5% (APC = 2.5; 95% CI: 1.9–3.1; *p* < 0.05) (Table 2, Table 3, Table 4 and Table 6).

**Figure 3:**
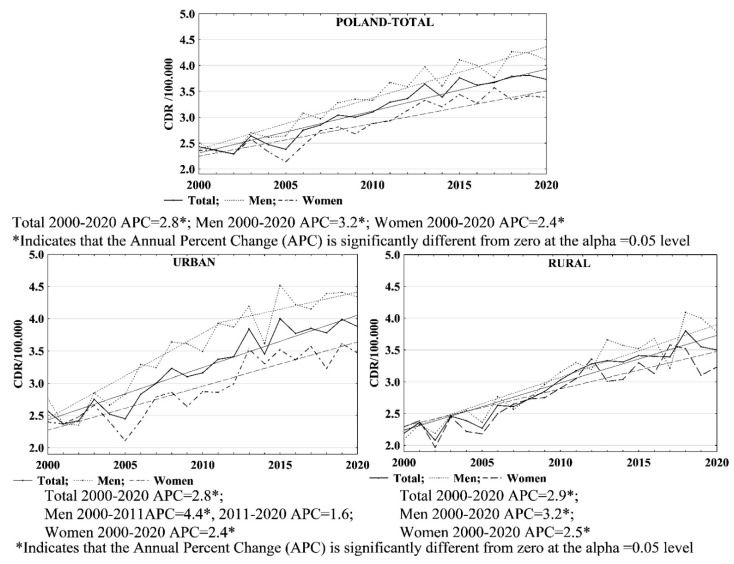
CDR due to skin melanoma by gender and place of residence in Poland in 2000–2020.

The values for crude death rates contributed by skin melanoma were higher for men than for women for all Polish residents, urban and rural, throughout the study period. Between 2000 and 2020, higher CDR values were recorded for urban residents compared to rural residents. Larger differences between CDR values for men and women were noted for urban residents (Figure 3).

In order to eliminate the impact of age on the observed differences in calculated CDR values, standardised death rate (SDR) values were calculated.

Between 2000 and 2020, the standardised death rates (SDR) due to skin melanoma in Poland per 100,000 population followed an upward trend. In the years 2000 and 2020, these rates were 3.60 and 4.03, respectively. In this period, the average annual percentage change (AAPC) in SDR values was 1.1% (AAPC = 1.1; 95% CI: 0.2–2.1; *p* < 0.05). In the year 2015, a significant change in the direction and rate of change regarding SDR values was observed. Between 2000 and 2015, SDR values increased at a rate of 1.9% (APC = 1.9; 95% CI: 1.2–2.5; *p* < 0.05), while between 2015 and 2020, they decreased at a rate of 1.0% (APC = −1.0; 95% CI: −4.3–2.4; *p* > 0.05). For men, SDR values increased from 4.45 in 2000 to 5.44 in 2020 (APC = 2.1; 95% CI: 1.6–2.6; *p* < 0.05). With regards to women, the SDR value in 2000 was 3.12 and in 2020 it was 3.10, while AAPC = 0.0% (AAPC = 0.0; 95% CI: −0.9–1.0; *p* > 0.05). Over the 21-year time period, the trend in terms of SDR values in the women group changed twice; between 2000 and 2005, the APC value was −2.4% (APC = −2.4; 95% CI: −5.0–0.2; *p* > 0.05); between 2005 and 2013, the SDR values increased by 2.8% per year (APC = 2.8; 95% CI: 1.2–4.5; *p* < 0.05); and then, between 2013 and 2020, they started to decrease again at a rate of 1.3% (APC = −1.3; 95% CI: −2.8–0.3; *p* > 0.5) (Table 2, Table 3, Table 4 and Table 7).

In the group of urban residents, the SDR values were the following: 3.56 in 2000 and 3.91 in 2020 (APC = 1.2; 95% CI: 0.7–1.6; *p* < 0.05). For men, the values were 4.57 in 2000 and 5.39 in 2020 (APC = 1.8; 95% CI: 1.2–2.5; *p* < 0.05), whereas for women, they were 2.94 in 2000 and 3.00 in 2020 (APC = 0.6; 95% CI: 0.0–1.3; *p* < 0.05) (Table 2, Table 3, Table 4 and Table 7).

In rural residents, the SDR values in 2000 and 2020 were 3.00 and 4.24, respectively (APC = 2.2; 95% CI: 1.7–2.6; *p* < 0.05). For men, the SDR values in 2000 and 2020 were 3.24 and 5.48, respectively (APC = 3.0; 95% CI: 2.4–3.5; *p* < 0.05). In female rural residents, the SDR value calculated for skin melanoma increased from 2.83 in 2002 to 3.29 in 2020. The growth rate of the SDR value for women over the entire study period was 1.5% (APC = 1.5; 95% CI: 0.9–2.1; *p* < 0.05) (Table 2, Table 3, Table 4 and Table 7).

**Figure 4:**
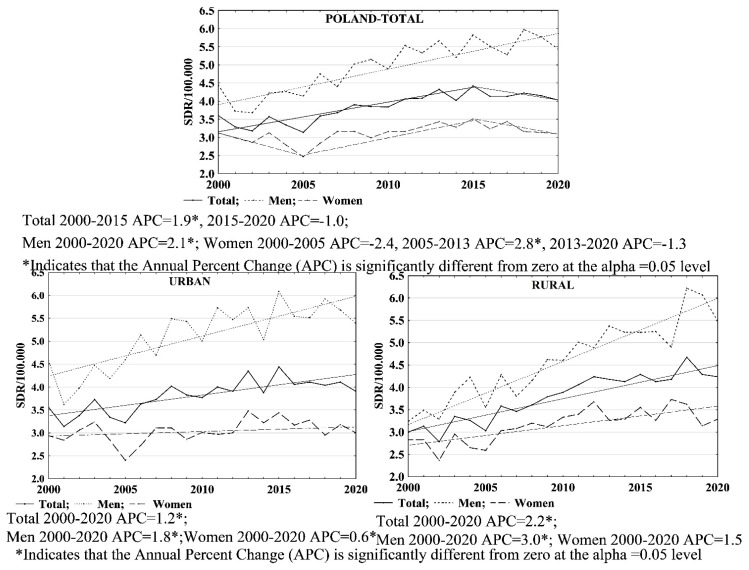
SDR due to skin melanoma by gender and place of residence in Poland in 2000–2020.

Values of standardised death rates due to skin melanoma were higher in the male group than in the female group, both for all Polish residents and for urban and rural residents. The mortality rates due to skin melanoma throughout the analysed 21-year period were similar for urban and rural residents. From 2016 onwards, more significant differences in mortality between urban and rural residents were observed, and rural residents were at a disadvantage. With regards to the male group, skin melanoma-related mortality was higher in urban residents than in rural residents for the majority of the analysed period. Yet, in the most recent period, i.e., between 2018 and 2020, the trend changed, putting rural residents at a disadvantage. With regards to women, the values of SDR rates were higher for rural residents throughout almost the entire study period (Figure 4).

## 4. Discussion

In Poland, as in other countries, an increase in mortality due to skin melanoma is evident [29]. This study provides detailed data on mortality due to skin melanoma in Poland in the first two decades of the 21st century. During this period, an upward trend in mortality was observed in the entire Polish population as well as in subpopulations of men and women as well as in urban and rural residents. The highest increase in the rate of mortality due to this neoplasm was observed in the male group as well as among rural residents. Increasing trends in the values in terms of mortality rates due to MM were higher for men than for women. The results of the study correspond to data obtained for 31 countries between the years 1985 and 2015: for men, skin melanoma-related mortality demonstrated an increasing trend, while in the female group, this trend was stable [29].

The increased incidence of skin melanoma and related mortality can be attributed to changes taking place in people’s outdoor activities and exposure to sunlight, especially related to tourism [30].

MM mortality increased with age, which is mainly due to an accumulation of risk factors for skin melanoma over a lifetime [31]. In the case of Polish citizens, the accumulation of risk factors of this type of cancer over a lifetime may be one of reasons for the increased cancer-related incidence and mortality because Poland has an aging society. In Poland, the percentage of people aged 65 or older increased from 12.2% in 2000 to 18.6% in 2020 [25]

As in other countries, in Poland, skin melanoma-related mortality in men is higher than in women. This difference was also noted for urban and rural residents and may be due to poorer sun protection behaviour among men and unfavourable biological conditions in the case of men, who are more prone to be affected by cancer. Additionally, men living in rural areas and working outdoors are exposed to solar radiation for longer periods [31,32]. Women, despite sunbathing more often than men, are more aware of the necessity of protection against sun radiation, and they also follow health campaigns concerning issues of prevention and the early detection of cancer [29]. Another reason for the higher mortality rate in men due to MM may be associated with the difference in the localisation of this cancer in men and women. In women, skin melanoma most often affects legs and arms, i.e., more visible areas, while in men, it occurs on the trunk [3]. The location of the tumour often determines its earlier detection [29].

The results of the analysis showed that in the last two years of the study period, a number of favourable changes in skin melanoma-related mortality were noted in Poland. Slight and statistically insignificant decreasing mortality trends were observed annually: a 1% decrease was noted for the whole Polish population as well as a decrease of 1.3% among women.

It can be assumed that the positive changes in mortality due to MM observed in the latter years of the analysed period may partly result from new drug treatments for malignant melanoma, such as the use of niwolumab, pembrolizumab and atezolizumab, and partly from increased awareness among the Polish population regarding the issue of skin melanoma, the causes of its occurrence and methods of its diagnosis. In 2006, Poland joined the European Euromelanoma prevention campaign, which was initiated in Belgium in 1999 [33].

The aim of the Euromelanoma campaign is to raise public awareness of the prevention, early diagnosis and treatment of skin cancer. As part of this campaign, meetings and press conferences are organised, information leaflets and posters are distributed and, most importantly, free examinations of nevi are carried out during the so-called “Melanoma Day”. The activities of the Euromelanoma Association are multidirectional, targeting the public, scientific and governmental sectors in each country. In Poland, a number of campaigns have been organised to raise public awareness of MM. In May of each year since 2010, the “Melanoma Awareness Week” has been organised. On that day, educational materials on this cancer are promoted. Other popular campaigns aiming to raise awareness of skin melanoma among the Polish public are: “It might be melanoma. Check It”, “I sunbathe safely”, “Naevus! Do I know it?”. As part of the third above-mentioned action, a programme on melanoma was developed for students in the final grade of primary school and all students from secondary schools. The programme includes a lesson plan, a leaflet and a poster for the teacher’s lesson as well as a video presentation for students. The film shows the students how to protect themselves against skin melanoma and how to monitor their skin properly. More and more often, the Polish mass media, its press, television and radio organisations, provide information on MM. Additionally, there are many internet websites dedicated to the problem of skin melanoma. Changing public awareness is a long-term process, and several years after the campaigns on skin melanoma were initiated in Poland, it is still difficult to assess whether and to what extent the minor beneficial changes in mortality, noted during the study and discussed earlier, are related to these campaigns.

A number of beneficial changes can be seen in the study results, and the mortality rates due to skin melanoma are many times lower than due to other malignancies [34,35,36,37,38,39,40]. However, the situation regarding skin melanoma-related mortality in Poland is poor.

Available comparative analyses for nine European countries (Germany, the Czech Republic, Poland, Denmark, Austria, Belgium, France, the Netherlands and Switzerland) have shown that the rate of increase in mortality rates due to skin melanoma in Poland between the years 1980 and 2012, in both men and women, was one of the highest among these countries [41].

The mortality to incidence ratio (MIR), indicating the effectiveness of prevention and treatment methods for skin melanoma, is 46% in Poland [3]. This result is the worst of all countries included in the study. It is worth noting that in Australia, where the incidence rate of skin melanoma is the highest, the MIR value is the lowest, i.e., 11%. Among European countries, the lowest MIR values are observed for Germany (11%), Luxembourg (12%), Denmark (12%) and Switzerland (12%).

Skin melanoma-related mortality in Poland is high. In the middle of the period 2000–2020, the disease contributed to roughly 80% of deaths [42]. Such a high mortality rate is mainly related to the late detection of the cancer. According to the SEER (surveillance, epidemiology and end results), the 5-year survival for stage I-II skin melanoma is as high as 99.4%; for stage III, it declines to 68.0%, and for stage IV, it is only 29.8% [8]. In Poland, the 5-year survival for this cancer calculated for patients diagnosed between the years 2003 and 2005 was 65.0% and was higher in women (71.3%) than in men (56.4%) [43]. It is worth pointing out that the 5-year survival for skin melanoma calculated for the years 2010–2014 was already 69.8%. However, this result was considerably worse than that observed in Germany (93.1%) or the United Kingdom (90.9%) [44].

Skin melanoma has a poor prognosis at the metastatic stage. The excision of the tumour at an early stage may result in complete remission. Therefore, the early detection of this tumour is highly important [45]. This does not appear to be difficult. Everyone can themselves, or with the help of others, notice changes that appear on their skin. However, Polish data regarding skin melanoma, particularly skin melanoma-related mortality, MIR values and survival rates, may indicate that patients visit a doctor too late, with an advanced-stage cancer which has already metastasised. All these factors often result in unsuccessful therapy. It is estimated that in Poland, of the 2500–3000 new cases of MM detected annually, 1500 are in the advanced or disseminated stage [46].

The most important modifiable risk factor for skin melanoma is UV radiation. This cancer should be fought by raising public awareness regarding the dangers of exposure to solar and artificial radiation, the importance of covering sun-exposed body areas with clothing, protecting them with anti-UV cosmetics and preventing particularly young children from sunbathing. It is important to make the public aware that self-tanning devices have been classified as Class I human carcinogens by the International Agency for Research on Cancer [31].

Any pigmented nevi that appear on the skin should be immediately diagnosed and monitored. For those with macular, pigmented nevi, self-examination of the skin should become a daily habit. Instant patient reactions to the occurrence of nevi and immediate visits to doctors will result in early diagnoses.

An online survey on the knowledge of skin melanoma conducted in a group of respondents aged 15–55 years in Poland in 2016 showed that the majority of the respondents (81%) have heard of skin melanoma and know what it is. However, the remaining percentage of the people appeared to have insufficient knowledge about this issue. Only every fifth surveyed person admitted to regularly and closely observing skin lesions. Additionally, only one person in five visited a dermatologist to check their moles. The majority of Poles (58%) who noticed worrying skin lesions on their body did not visit a doctor so as to be diagnosed [47].

Primary care physicians and dermatologists should play an increasingly important role in the early diagnosis of MM. Worrying skin lesions should be routinely assessed with dermatoscopes and videodermatoscopes, which will allow doctors to detect the early signs of the disease, long before clinical changes occur. Innovative technologies, such as whole-body photography and multispectral imaging, improve accuracy and reliability in the detection of early melanoma [4,48].

A reduction in skin melanoma-related mortality is also possible through the introduction of screening examinations, particularly in groups at high risk of MM. Screening examinations primarily contribute to the early detection of dangerous skin lesions, which, in turn, allows for the implementation of appropriate treatment methods. Estimates made for the German population reveal that a 20% participation rate in screening examinations, conducted every two years among people aged 35–85 years, would contribute to a 45% decrease in skin melanoma-related mortality within two decades [41].

The effects of widespread prevention efforts in reducing mortality due to MM should be viewed with hope and optimism. Increased prevention efforts halted upward trends in skin melanoma-related mortality in Sweden and Spain [49,50], Italy [51] and, above all, in Australia, the country with the highest cancer incidence and mortality rates [30,52,53].

## 5. Conclusions

Skin melanoma is a serious public health problem in Poland. Mortality rates due to this neoplasm in the first two decades of the 21st century show increasing trends. They are higher in men than in women, both in urban and rural residents. The early diagnosis of melanoma is a public health priority. Primary and secondary prevention is highly important in reducing skin melanoma-related mortality. It is essential to target groups at high risk of developing skin melanoma as well as those who are affected by the highest mortality rate (particularly the elderly, men and rural residents), to carry out screening tests, to implement new prevention programmes and expand those which have been previously introduced. These tasks are a challenge for the government, who is supposed to take decisions in this area on the basis of projects developed in various ministries, primarily on health or education.

## Figures and Tables

**Table 1:** Mean age of people who died due to skin melanoma by gender and place of residence in Poland in 2000, 2010 and 2020.

Year	Place of Residence								
	Poland Total			Urban			Rural		
	Total	Men	Women	Total	Men	Women	Total	Men	Women
2000	62.4	61.7	63.0	62.4	61.7	63.0	63.0	61.0	64.9
2010	67.0	65.9	68.3	67.0	65.9	68.3	66.4	63.8	69.2
2020	72.4	70.8	74.2	72.4	70.8	74.2	71.7	67.6	76.3

**Table 2:** Number of deaths, CDR per 100,000 and SDR per 100,000 due to skin melanoma in Poland by gender in 2000, 2005, 2010, 2015 and 2020.

Year	Total			Men			Women		
	*n*	CDR	SDR	*n*	CDR	SDR	*n*	CDR	SDR
2000	928	2.43	3.60	463	2.50	4.45	465	2.36	3.12
2005	909	2.38	3.14	488	2.64	4.14	421	2.14	2.47
2010	1188	3.10	3.84	618	3.33	4.89	570	2.88	3.16
2015	1447	3.76	4.42	765	4.11	5.82	682	3.44	3.51
2020	1427	3.73	4.03	760	4.11	5.44	667	3.38	3.10

**Table 3:** Number of deaths, CDR per 100,000 and SDR per 100,000 due to skin melanoma of urban residents in Poland by gender in 2000, 2005, 2010, 2015 and 2020.

Year	Total			Men			Women		
	*n*	CDR	SDR	*n*	CDR	SDR	*n*	CDR	SDR
2000	608	2.57	3.56	311	2.76	4.57	297	2.40	2.94
2005	575	2.45	3.22	315	2.83	4.59	260	2.11	2.40
2010	736	3.16	3.77	384	3.49	5.00	352	2.87	3.01
2015	926	4.00	4.44	497	4.52	6.08	429	3.52	3.44
2020	889	3.88	3.91	471	4.34	5.39	418	3.47	3.00

**Table 4:** Number of deaths, CDR per 100,000 and SDR per 100,000 due to skin melanoma of rural residents in Poland by gender, 2000, 2005, 2010, 2015 and 2020.

Year	Total			Men			Women		
	*n*	CDR	SDR	*n*	CDR	SDR	*n*	CDR	SDR
2000	320	2.19	3.00	152	2.09	3.24	168	2.29	2.83
2005	334	2.27	3.03	173	2.36	3.55	161	2.18	2.59
2010	452	3.03	3.89	234	3.15	4.60	218	2.90	3.33
2015	521	3.41	4.29	268	3.52	5.23	253	3.30	3.55
2020	538	3.50	4.24	289	3.78	5.48	249	3.23	3.29

**Table 5:** Number of deaths, CDR values and SDR values due to skin melanoma by age and sex in Poland in 2000, 2010 and 2020.

Age Group	Year								
	2000			2010			2020		
	*n*	CDR	SDR	*n*	CDR	SDR	*n*	CDR	SDR
Total									
0–25	11	0.08	0.08	3	0.03	0.02	1	0.01	0.01
25–49	201	1.45	1.42	144	1.04	1.13	125	0.88	0.89
50–69	355	4.78	4.84	482	5.11	5.38	425	4.34	4.11
70+	361	11.79	13.55	559	14.69	15.09	876	18.98	19.28
Men									
0–24	5	0.07	0.07	3	0.05	0.05	0	0.00	0.00
25–49	109	1.57	1.58	78	1.11	1.23	77	1.07	1.09
50–69	186	5.44	5.58	281	6.34	6.76	272	5.85	5.65
70+	163	15.18	17.96	256	18.85	19.87	411	23.86	26.13
Women									
0–24	6	0.09	0.09	0	0.00	0.00	1	0.02	0.02
25–49	92	1.33	1.30	66	0.96	1.05	48	0.68	0.69
50–69	169	4.22	4.24	201	4.02	4.21	153	2.97	2.73
70+	198	5.14	11.43	303	6.84	12.52	465	8.17	15.54
Urban									
0–24	8	0.10	0.09	1	0.02	0.01	1	0.02	0.02
25–49	133	1.51	1.45	81	0.95	1.07	75	0.89	0.91
50–69	233	4.77	4.87	307	4.98	5.2	251	4.18	3.88
70+	234	13.06	14.85	347	14.70	15.1	562	18.44	18.8
Men									
0–24	3	0.07	0.07	1	0.03	0.03	0	0.00	0.00
25–49	70	1.32	1.58	44	1.04	1.19	40	0.95	0.95
50–69	126	5.70	5.90	174	6.25	6.70	155	5.66	5.39
70+	112	18.19	20.75	165	19.77	20.93	276	24.51	26.60
Women									
0–24	5	0.12	0.12	0	0.00	0.00	1	0.04	0.04
25–49	63	1.39	1.32	37	0.86	0.95	35	0.83	0.86
50–69	107	4.00	4.05	133	3.93	4.02	96	2.94	2.61
70+	122	10.37	11.75	182	11.93	12.06	286	14.89	14.68
Rural									
0–24	3	0.05	0.06	2	0.04	0.04	0	0.00	0.00
25–49	68	1.35	1.39	63	1.18	1.29	50	0.87	0.90
50–69	122	4.67	4.71	175	5.33	5.65	174	4.60	4.50
70+	127	9.58	9.86	212	14.43	14.58	314	20.03	20.12
Men									
0–24	2	0.07	0.08	2	0.08	0.07	0	0.00	0.00
25–49	39	1.46	1.49	34	1.22	2.15	37	1.25	1.97
50–69	60	4.83	4.95	107	6.52	7.78	117	6.13	7.30
70+	51	10.61	11.02	91	17.09	14.00	135	22.64	21.78
Women									
0–24	1	0.04	0.04	0	0.00	0.00	0	0.00	0.00
25–49	29	1.21	1.27	29	1.12	1.77	13	0.47	0.80
50–69	62	4.53	4.55	68	4.14	5.09	57	3.04	4.24
70+	76	9.00	8.89	121	12.92	10.72	179	18.43	14.28

**Table 6:** Time trends of CDR due to skin melanoma by gender and place of residence in Poland in 2000–2020—results of joint regression analysis.

Place of Residence/Gender	Number of Joinpoints	Years	APC	95% CI	AAPC	95% CI
Poland						
Men	0	2000–2020	3.2 *	(2.7; 3.7)		
Women	0	2000–2020	2.4 *	(2.0; 2.9)		
Total	0	2000–2020	2.8 *	(2.4; 3.2)		
Urban						
Men	1	2000–2011 2011–2020	4.4 * 1.6	(2.9; 5.9) (−0.4; 3.6)	3.1 *	(2.0; 4.3)
Women	0	2000–2020	2.4 *	(1.8; 3.0)		
Total	0	2000–2020	2.8 *	(2.3; 3.3)		
Rural						
Men	0	2000–2020	3.2 *	(2.7; 3.7)		
Women	0	2000–2020	2.5 *	(1.9; 3.1)		
Total	0	2000–2020	2.9 *	(2.4; 3.3)		

* *p* < 0.05.

**Table 7:** Time trends of SDR due to skin melanoma by gender and place of residence in Poland in 2000–2020—results of the joinpoint regression analysis.

Place of Residence/Gender	Number of Joinpoints	Years	APC	95% CI	AAPC	95% CI
Poland						
Men	0	2000–2020	2.1 *	(1.6; 2.6)		
Women	2	2000–2005 2005–2013 2013–2020	−2.4 2.8 * −1.3	(−5.0; 0.2) (1.2; 4.5) (−2.8; 0.3)	0.0	(−0.9; 1.0)
Total	1	2000–2015 2015–2020	1.9 * −1.0	(1.2; 2.5) (−4.3; 2.4)	1.1 *	(0.2; 2.1)
Urban						
Men	0	2000–2020	1.8 *	(1.2; 2.5)		
Women	0	2000–2020	0.6 *	(0.0; 1.3)		
Total	0	2000–2020	1.2 *	(0.7; 1.6)		
Rural						
Men	0	2000–2020	3.0 *	(2.4; 3.5)		
Women	0	2000–2020	1.5 *	(0.9; 2.1)		
Total	0	2000–2020	2.2 *	(1.7; 2.6)		

* *p* < 0.05.

## Data Availability

The data presented in this study are available on request from the corresponding author.

## Supplementary Materials

The following supporting information can be downloaded at: https://www.mdpi.com/article/10.3390/ijerph192316118/s1. Table S1: Mean age of people who died due to skin melanoma by gender and place of residence in Poland in 2000–2020. Table S2: Number of deaths, CDR per 100,000, SDR per 100,000 due to skin melanoma in Poland by gender in 2000–2020. Table S3: Number of deaths, CDR per 100,000, SDR per 100,000 due to skin melanoma of urban residents in Poland by gender in 2000–2020. Table S4: Number of deaths, CDR per 100,000, SDR per 100,000 due to skin melanoma of rural residents in Poland by gender, 2000–2020.
