# Supplementary material for: Mortality Trends Due to Skin Melanoma in Poland in the Years 2000–2020
Source: Int J Environ Res Public Health. 2022 Dec 2;19(23):16118. doi: 10.3390/ijerph192316118 (PMC9739595; doi:10.3390/ijerph192316118)
Supplement: Supplementary file 1 [file ijerph-19-16118-s001.zip › ijerph-2027278-supplementary.pdf]

Table S1. Mean age of people who died due to skin melanoma by gender and place of residence in Poland in 2000-2020.

Year	Place of residence								
	Poland-total			Urban			Rural		
	Total	Men	Women	Total	Men	Women	Total	Men	Women
2000	62.4	61.7	63.0	62.4	61.7	63.0	63.0	61.0	64.9
2001	61.1	59.2	62.9	61.1	59.2	62.9	61.0	59.5	62.5
2002	63.0	61.6	64.2	63.0	61.6	64.2	62.3	60.0	64.9
2003	63.1	61.8	64.3	63.1	61.8	64.3	63.5	60.9	66.2
2004	62.8	60.8	64.8	62.8	60.8	64.8	63.5	62.1	65.0
2005	64.3	63.8	64.9	64.3	63.8	64.9	63.2	59.7	67.0
2006	64.2	63.8	64.7	64.2	63.8	64.7	64.0	61.5	66.7
2007	64.3	63.4	65.3	64.3	63.4	65.3	65.9	61.8	69.9
2008	66.6	65.4	67.9	66.6	65.4	67.9	65.9	63.1	68.6
2009	66.5	65.0	68.3	66.5	65.0	68.3	67.1	64.1	70.3
2010	67.0	65.9	68.3	67.0	65.9	68.3	66.4	63.8	69.2
2011	67.4	66.5	68.5	67.4	66.5	68.5	67.4	65.5	69.6
2012	67.7	67.3	68.2	67.7	67.3	68.2	68.2	64.3	71.8
2013	68.8	67.1	70.5	68.8	67.1	70.5	66.8	63.6	70.7
2014	69.6	68.7	70.5	69.6	68.7	70.5	67.6	64.6	71.0
2015	69.5	67.8	71.4	69.5	67.8	71.4	69.8	65.9	74.0
2016	70.5	68.9	72.3	70.5	68.9	72.3	68.8	65.2	73.0
2017	71.4	70.0	72.8	71.4	70.0	72.8	72.0	68.9	74.8
2018	71.9	71.2	72.8	71.9	71.2	72.8	71.4	68.6	74.6
2019	72.3	70.9	73.9	72.3	70.9	73.9	71.5	68.8	74.8
2020	72.4	70.8	74.2	72.4	70.8	74.2	71.7	67.6	76.3

Table S2. Number of deaths, CDR per 100,000, SDR per 100,000 due to skin melanoma in Poland by gender in 2000-2020.

Year	Total			Men			Women		
	n	CDR	SDR	n	CDR	SDR	n	CDR	SDR
2000	928	2.43	3.60	463	2.50	4.45	465	2.36	3.12
2001	904	2.36	3.29	436	2.35	3.72	468	2.37	2.99
2002	876	2.29	3.18	424	2.29	3.68	452	2.29	2.86
2003	1008	2.64	3.57	499	2.70	4.22	509	2.58	3.13
2004	943	2.47	3.34	483	2.61	4.26	460	2.33	2.79
2005	909	2.38	3.14	488	2.64	4.14	421	2.14	2.47
2006	1050	2.75	3.59	568	3.08	4.75	482	2.45	2.84
2007	1086	2.85	3.68	547	2.97	4.40	539	2.74	3.16
2008	1158	3.04	3.90	604	3.28	5.02	554	2.81	3.17
2009	1146	3.00	3.85	617	3.35	5.15	529	2.68	2.99
2010	1188	3.10	3.84	618	3.33	4.89	570	2.88	3.16
2011	1268	3.29	4.06	685	3.67	5.53	583	2.93	3.16
2012	1293	3.36	4.08	670	3.59	5.33	623	3.13	3.29
2013	1402	3.64	4.32	740	3.97	5.66	662	3.33	3.43
2014	1306	3.39	4.02	670	3.60	5.20	636	3.20	3.28

2015	1447	3.76	4.42	765	4.11	5.82	682	3.44	3.51
2016	1393	3.62	4.13	744	4.00	5.51	649	3.27	3.24
2017	1409	3.67	4.13	700	3.76	5.28	709	3.57	3.43
2018	1456	3.79	4.22	793	4.27	5.97	663	3.34	3.16
2019	1464	3.81	4.15	788	4.24	5.77	676	3.41	3.14
2020	1427	3.73	4.03	760	4.11	5.44	667	3.38	3.10

Table S3. Number of deaths, CDR per 100,000, SDR per 100,000 due to skin melanoma of urban residents in Poland by gender in 2000-2020.

Year	Total			Men			Women		
	n	CDR	SDR	n	CDR	SDR	n	CDR	SDR
2000	608	2.57	3.56	311	2.76	4.57	297	2.40	2.94
2001	559	2.37	3.14	266	2.37	3.62	293	2.37	2.84
2002	571	2.42	3.38	264	2.35	3.98	307	2.48	3.06
2003	648	2.75	3.73	319	2.85	4.47	329	2.66	3.24
2004	592	2.52	3.34	296	2.66	4.19	296	2.40	2.84
2005	575	2.45	3.22	315	2.83	4.59	260	2.11	2.40
2006	662	2.83	3.63	365	3.29	5.13	297	2.42	2.73
2007	700	3.00	3.73	358	3.24	4.69	342	2.79	3.11
2008	752	3.23	4.02	402	3.64	5.49	350	2.86	3.11
2009	721	3.10	3.83	398	3.61	5.43	323	2.64	2.86
2010	736	3.16	3.77	384	3.49	5.00	352	2.87	3.01
2011	787	3.37	4.00	436	3.93	5.73	351	2.86	2.97
2012	795	3.41	3.91	428	3.87	5.47	367	2.99	3.00
2013	894	3.84	4.35	462	4.19	5.73	432	3.53	3.48
2014	801	3.45	3.88	398	3.61	5.03	403	3.30	3.22
2015	926	4.00	4.44	497	4.52	6.08	429	3.52	3.44
2016	872	3.77	4.06	463	4.22	5.54	409	3.36	3.17
2017	889	3.85	4.11	455	4.15	5.51	434	3.57	3.28
2018	874	3.78	4.04	481	4.39	5.92	393	3.23	2.95
2019	920	3.99	4.11	482	4.41	5.68	438	3.61	3.18
2020	889	3.88	3.91	471	4.34	5.39	418	3.47	3.00

Table S4. Number of deaths, CDR per 100,000, SDR per 100,000 due to skin melanoma of rural residents in Poland by gender, 2000-2020.

Year	Total			Men			Women		
	n	CDR	SDR	n	CDR	SDR	n	CDR	SDR
2000	320	2.19	3.00	152	2.09	3.24	168	2.29	2.83
2001	345	2.36	3.13	170	2.33	3.49	175	2.39	2.83
2002	305	2.08	2.78	160	2.19	3.29	145	1.97	2.37
2003	360	2.46	3.35	180	2.47	3.89	180	2.45	2.95
2004	351	2.39	3.26	187	2.55	4.23	164	2.22	2.65
2005	334	2.27	3.03	173	2.36	3.55	161	2.18	2.59
2006	388	2.63	3.58	203	2.77	4.28	185	2.50	3.03
2007	386	2.61	3.46	189	2.57	3.80	197	2.65	3.08
2008	406	2.73	3.61	202	2.74	4.15	204	2.73	3.20
2009	425	2.85	3.79	219	2.96	4.62	206	2.75	3.12
2010	452	3.03	3.89	234	3.15	4.60	218	2.90	3.33
2011	481	3.17	4.06	249	3.30	5.01	232	3.05	3.40
2012	498	3.28	4.24	242	3.19	4.89	256	3.36	3.68
2013	508	3.33	4.18	278	3.66	5.37	230	3.01	3.26

2014	505	3.31	4.13	272	3.57	5.23	233	3.04	3.29
2015	521	3.41	4.29	268	3.52	5.23	253	3.30	3.55
2016	521	3.40	4.13	281	3.68	5.25	240	3.13	3.26
2017	520	3.39	4.18	245	3.21	4.90	275	3.58	3.73
2018	582	3.80	4.67	312	4.09	6.22	270	3.52	3.62
2019	544	3.55	4.29	306	4.00	6.07	238	3.10	3.14
2020	538	3.50	4.24	289	3.78	5.48	249	3.23	3.29
